# Hydrothermal synthesis of rGO and MnCoS composite for enhanced supercapacitor application

**DOI:** 10.1038/s41598-024-77245-5

**Published:** 2024-10-27

**Authors:** M. Manikandan, T. Prasankumar, E. Manikandan, E. Papanasam, K. Ramesh, S. Ramesh

**Affiliations:** 1 Centre for Advanced Materials and Innovative Technologies, Vellore Institute of Technology, Chennai, 600127 India; 2 School of Electronics Engineering, Vellore Institute of Technology, Chennai, 600127 India; 3 Department of Physics, Centre for Ionics Universiti Malaya, Universiti Malaya, Kuala Lumpur, 50603 Malaysia; 4 Institute of Power Engineering, Universiti Tenaga Nasional (UNITEN), Kajang, 43000 Selangor Malaysia

**Keywords:** rMCS//rGO, Asymmetric supercapacitor, Ternary metal sulfides, Energy density and power density, Energy science and technology, Nanoscience and technology

## Abstract

Nanostructured materials incorporating transition metal sulfides have demonstrated considerable potential across various applications, particularly in the realms of energy production and storage. Sulfide-based material preparation is a challenging and costly procedure that requires a high temperature and reducing atmosphere. This work reports that manganese cobalt sulfide (MCS) and reduced graphene oxide composite manganese cobalt sulfide (rMCS) were successfully prepared through a hydrothermal method. Various characterization techniques were employed to analyze the prepared materials, including X-ray diffraction, field emission scanning electron microscopy, transmission electron microscopy, Brunauer-Emmett-Teller analysis, and X-ray photoelectron spectroscopy. In a three-electrode system, MCS and rMCS electrodes exhibit an excellent specific capacitance of 1695 and 1925 F g^−1^ at 1 A g^−1^ current density respectively. MCS delivers the capacitance retention of 99% and rMCS exhibits the capacitance retention of 100% capacitance retention over 5000 consecutive cycles. The constructed asymmetric supercapacitor electrode (rMCS//rGO) exhibits the energy and power density of 64 Wh kg^−1^ at 799 W kg^−1^, respectively with outstanding cyclic stability of 97.4% even after 10,000 cycles. The exceptional electrochemical properties of MCS with rGO composite electrode indicate that they would make an outstanding electrode material for cutting-edge energy storage devices.

## Introduction

Supercapacitors (SCs) are popular in electric vehicles and wearable electronics for their high energy density and cycling stability. Still, in comparison to rechargeable batteries, it has lower energy density. Supercapacitors contain a extensive vary of applications due to their ability to deliver higher energy density while maintaining power density^1–3^. SCs possess distinct attributes, including high power density, cycling stability, low equivalent series resistance, safe operation conditions, and rapid charge/discharge rates, rendering them promising candidates as energy storage devices. SCs are becoming more prevalent in hybrid electric vehicles, portable electronics and emergency power systems. SCs are classified into two types; they are electrochemical double-layer capacitors (EDLCs) and pseudocapacitors (PCs) based on their energy storage mechanisms^4–7^. In EDLCs, energy storage and conversion occur through charge separation at the Helmholtz layer. The most commonly used materials for double-layer electrodes are carbon-based. Pseudocapacitive materials, such as metal oxides or conducting polymers, store energy via rapid and reversible redox reactions occurring at the surface^8–12^. EDLCs typically exhibit higher power densities and ultra-long cycling capabilities, whereas PCs offer superior energy densities but lesser power densities and cycling stability in view of reduced conductivity. Transition metal oxides and sulfides serve as highly efficient active materials owing to their varied oxidation states, cost-effectiveness, and enhanced energy storage capacity^13–15^. Transition metal sulfides (TMSs) are gaining popularity as energy storage devices in behalf of their inexpensive and unique properties, including high specific capacity, improved reactivity and conductivity, and low electronegativity of sulfur elements. Sulfur’s low electronegativity can shorten the ionic diffusion path in nanoarchitecture, leading to improved electrochemical performance and mechanical integrity. The CoS_x_ and MnS_x_ materials offer exceptional performance, cost-effectiveness, environmental friendliness, and flexibility. Manganese is one of the more abundant transition metals in the Earth’s crust, making it a relatively sustainable option for large-scale energy storage applications. Cobalt, while less abundant, can still offer environmental benefits when its sourcing is optimized. Both elements are less hazardous than other heavy metals traditionally used in energy storage devices, like lead or cadmium, contributing to a reduced environmental impact^16–18^. Transition metal sulfides (TMSs) are becoming increasingly favored as electrode materials for supercapacitors, thanks to their enhanced conductivity, redox capabilities, lower electronegativity, and catalytic properties, such as increased electrochemical surface area and presence of electroactive redox sites, which surpass those of transition metal oxides. Sulphospinel materials, particularly those containing manganese (Mn) and cobalt (Co), have gained traction as cathode materials for supercapacitors due to their affordability, wide availability, ease of processing, and significant capacity contribution^2,19–21^.

To be thought about for practical applications, ternary sulfide electrodes must improve their rate performance and cycling stability while maintaining energy density. TMSs, including MnCo_2_S_4_, NiCo_2_S_4_, ZnCo_2_S_4_, and CuCo_2_S_4_, exhibit higher electrochemical reactivity and specific capacity due to the cooperative effect of two metal ions. Mandal et al. reported electrodeposition binder-free MnCoS nanosheets for high-performance supercapacitors with density of energy and power 105 W h kg^−1^ and 72 kW kg^−1^^22^. Fabricated MnCo_2_S_4_ nanowires showed excellent capacitance retention (rate capabilities, long cycling) of 96.5% even 3000 rotation at the density of current 2 A g^−1^^23^. A novel quasi-solid-state supercapacitor incorporating rGO/Ni_1_Co_1_S and rGO/Fe_2_O_3_ electrodes, along with an inorganic/organic composite solid electrolyte, was fabricated to assess its energy storage capabilities. Subsequently, sensible LED lighting arrangements employing series devices and addressing self-discharge (SDC) were implemented^24^. The Zn: Co (1:2) electrode material produced at 50 °C has a specific capacities of 1134.7 F g^−1^ at 1 A g^−1^, as well as cyclic stability of 81% like of 1 to 20 A g^−1^). After 6000 charging-discharging cycles, the capacity does not decrease, making it an ideal anode for supercapacitors^25^. CuCoS//AC made asymmetric supercapacitor device displays the cyclic stability of 126% for the current density of 25 mA cm^− 2^ even after 2000 cycles^26^. Cheng et al. prepared CuCoS nanoarrays through hydrothermal method; the prepared electrode shows the energy density of 59.96 Wh kg^−1^ at the power density of 320 W kg^−1^^27^. NiCoS was grown on porous carbon nanofibers achieved the specific capacitance of 334.7 mAh g^−1^ at current density of 2 A g^−1^ have been reported by Chen et al.^28^.

Graphene, composed of single or more layers of 2D carbon sheets, has garnered significant interest. Therefore, utilizing it as a conductive substrate is a feasible option. Graphene-based nanohybrids include garnered interest due to their distinct physicochemical properties when combined with 2D nanosheets. Incorporating graphene into electrode materials offers unique advantages such as fast electron transfer, strong structural stability, and numerous electrochemical active sites^29,30^. Reduced graphene oxide (rGO), a form of nanostructured carbon, finds widespread function as an electrode material in SCs due to this outstanding electrical properties, chemical stability, larger specific surface area, improved mesopore size distribution, and enhanced conductivity^31,32^. Incorporating rGO into composites enhances electrode stability and conductivity. TMS have been identified as a category of pseudocapacitive materials in numerous studies. Cobalt sulfides exhibit significant electronic conductivity and flexibility, which can be attributed to the electronegativity of sulphur and the reduced optical band gap of Ni-Co. Integrating rGO with TMS material, has yielded better electrochemical performance, encompassing both electric double-layer capacitance (EDLC) and pseudocapacitance behavior. Composites combining rGO with metal sulfides present distinctive properties and heightened performance when compared to their individual constituents^32,33^. Li et al., proposed NiCo_2_S_4_/rGO//AC ASC own an extensive voltage window of 1.5 V, elevated energy of 24.4 W h kg^−1^ and power of 750 W kg^−1^ in 2 mol/L KOH^32^. ZnCoS/rGO//AC achieved 92% cyclic retention up to 10,000 cycles at 5 A g^−1^, resulting in density of energy & power is 29 Wh kg^−1^ and 2215 W kg^−1^^3^. The ASC (Ni_1.64_Co_2.40_S_4_/rGO//AC) has outstanding electrochemical performance, among cyclic stability of 92.6% after 10,000 cycles with an density of is energy and power 30.4 Wh kg^−1^ and 10 kW kg^−1^^33^. Chen et al. fabricated MnCoS/rGO//AC electrode delivered an density of energy is 76.7 Wh kg^−1^ and density of power is 800 W kg^−1^ among a cyclic capacitance of 100% up to 6000 cycles^34^. MnCo_2_S_4_ doped graphene with N and S elements achieves an outstanding specified capacities of 1324 F g^−1^ at 1 A g^−1^ with excellent conductivity also cyclic performance have been reported by Wang et al.,^35^. Manh et al. fabricated rGO/NiCoS//rGO/NiCoS symmetric supercapacitor devices demonstrated the energy at power density of 47.2 Wh kg^−1^ at 1.6 kW kg^−1^, 98% retention upto 5000 cycles^36^. Hybrid electrodes containing MnCoS and rGO are expected to perform well electrochemically. CoMnS@rGO composite electrode shows the energy and power density of 50.8 Wh kg^−1^ and 3440 W kg^−1^ among a cyclic capacitance of 92% up to 20,000 cycles^37^.

**Scheme 1:**
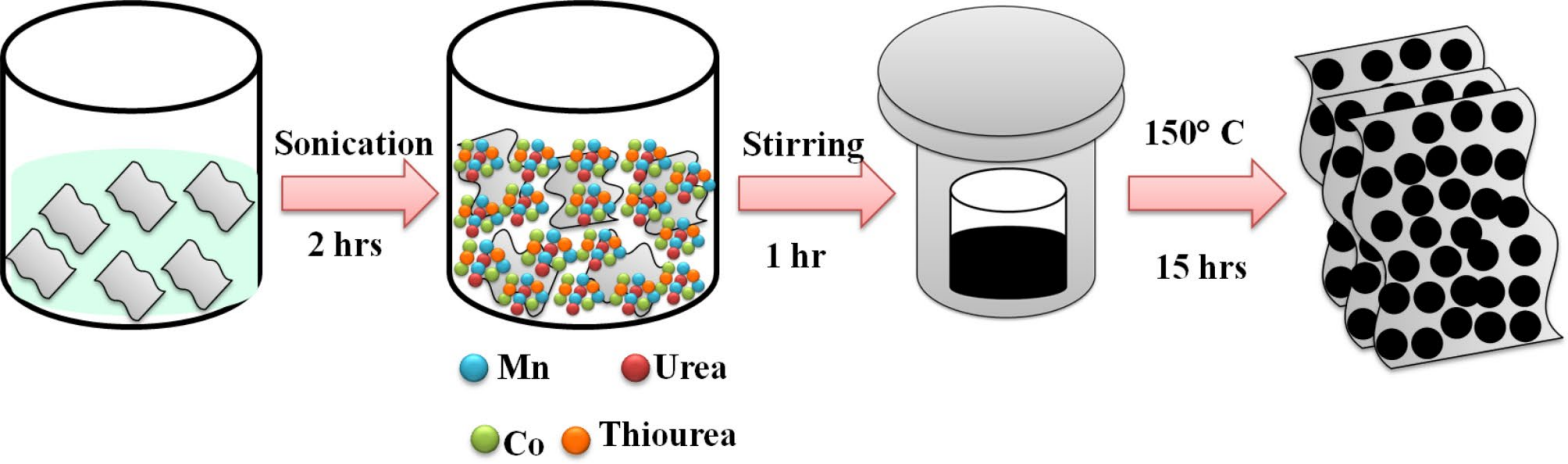
Graphic demonstration for the growth of rGO composite MnCoS nanoparticles.

In this report, hydrothermal method was used to synthesize MCS and rMCS for efficient supercapacitor applications. rGO-MCS composites offer a promising combination of environmental and economic benefits, particularly due to the abundance and low toxicity of manganese, the scalability of graphene production, and the high performance of the composite. However, the ethical and environmental concerns surrounding cobalt, along with the need for sustainable graphene production methods, should be addressed to maximize the sustainability and cost-effectiveness of these materials in energy storage applications. The prepared materials reveal the capacitance retention of 99% and 100% up to 5000 cycles with the capacitance of 1695 and 1925 F g^−1^ at 1 A g^−1^ density of current correspondingly, for MCS with rMCS. The fabricated asymmetric supercapacitor device delivers high density of energy and power is 64 Wh kg^−1^ and 799 W kg^−1^ with the specific capacities of 180 Fg^−1^ at the density of current 1 Ag^−1^. Scheme 1 illustrates the hydrothermal synthesis of MnCoS and MnCoS/rGO composite for asymmetric supercapacitor devices. MnCoS/rGO//AC demonstrated superior super capacitive performance, including high specific capacitance, energy density, and cyclic stability, making them ideal for energy storage applications.

## Experimental procedure

Manganese nitrate hexahydrate (Mn(NO_3_)_2_.6H_2_O), cobalt nitrate hexahydrate (Co(NO_3_)_2_.6H_2_O), urea (CH_4_N_2_O), thiourea (CH_4_N_2_S), and graphene oxide (GO) be bought from Merck as well as deionized water obtained from a Milli-Q-ultrapure water system (18.2 MΩ cm^−1^) for the experiment.

### Synthesis of MCS and rMCS

Manganese cobalt sulfide is produced through the hydrothermal methods. Using a standard procedure, 16 mmol CH_4_N_2_O, 12 mmol Co(NO_3_)_2_.6H_2_O, and 6 mmol Mn(NO_3_)_2_.6H_2_O were suspended in 80 ml of double distilled water while being stirred magnetically. The previously mentioned solution was then mixed with 24 mmol of CH_4_N_2_S for 1 h at room temperature. The solvent was carried out at 150 °C for 15 h in a 100 ml stainless steel reactor. After the autoclave had cooled to room temperature, the attained produce be rinsed a number of times using double distilled water and ethanol and vacuum-dried at 60 °C^38^. The sample is coded as MCS. rGO/MCS samples were prepared under same condition to which 30 mg of GO in a specific amount of double distilled water, the mixture was subjected to ultrasonication for 2 h The sample is coded as rMCS.

### Materials characterizations

X-ray diffraction (XRD) patterns were obtained by ARL EQUINOX 3000 with Cu Kα radiation (1.5406Å ) range from 20° to 80°, the Raman spectrum was acquired using a micro-Raman spectrometer (RENISHAW, UK) ready with a laser excitation source emitting light at a wavelength of 532 nm. Surface area of the electrodes was determined by BET (Brunauer-Emmett-Teller) analysis. The experiment utilized a Micromeritics ASAP 2020 analyzer to conduct N2 adsorption-desorption tests. X-ray photoelectron spectroscopy (XPS, Physical Electronics) was used to analyse the elemental composition and oxidation states of metal ions. High-resolution transmission electron microscopy (HRTEM; JEOL Japan, JEM-2100 plus) and field-emission scanning electron microscopy (FESEM) were employed for morphological analysis of the samples.

### Electrochemical measurement

Cyclic voltammetry (CV), galvanostatic charge-discharge (GCD), and electrochemical impedance spectroscopy (EIS) be utilized to assess the electrochemical performance of the electrode materials by using Squidat (Admiral) electrochemical workstation in a 3.5 M KOH aqueous electrolyte. The rGO/MCS composite, polyvinylidene fluoride (PVDF) as a binder, and conductive additive (super black) were mixed in 80:15:5 ratios to fabricate the working electrode. Some specific amount of active material, conductive additive and binder was weighted and grounded in a mortar with a solvent (NMP). The mixture was then agitated to create a uniform slurry, which was then uniformly applied on cleaned carbon cloth (1 × 2 cm). The mixture was then dried for 12 h at 80 °C in an oven. The weight of the substance that is active (2 mg) was determined. CV, GCD and EIS experiments were carrying out using 3.5 M KOH as the electrolyte. CV curves were obtained within a voltage range from 0 to 0.6 V, with scan speeds range from 5 to 50 mVs^−1^. GCD curves were gained from various current densities from 1 to 5 A g^−1^ with a potential of 0 to 0.6 V. To perform the electrochemical impedance spectroscopic (EIS) analysis in the frequency vary of 0.1 Hz to 100 kHz, alternate current (AC) with a bias voltage of 5 mV was also utilized. The specified capacities of the electrode materials were calculate using the follow equation^39^

1 $${\text{C}}={\text{I}}\Delta {\text{t/m}}\Delta {\text{v}}$$

where, m denotes the mass of the active material (g), Δt represents the discharge process (s), I represents the discharge process in the GCD curve (A g^−1^), and C denotes specified capacitance of the electrode materials (F g^−1^). Δv represents the potential range (v).

Using rMCS as the positive electrode and rGO as the negative electrode, an asymmetric supercapacitor (ASC) device with cellulose membranes as a separator was designed in KOH solution. ASC benefits from having a higher specific capacity and cell potential. To enhance ASC performance, it is imperative to utilize the following relationship to continue charge equilibrium among the positive and negative electrodes: q^+^ = q^-^, where q + and q-, respectively, represent the stored charges on the positive and negative electrode surfaces. The quantity of charge stored on the electrodes is dictated by the mass (m) and capacity (C) of the electrodes. Therefore, the mass stability equation can be formulated as follows:^40^

2 $${\text{q}}_{+} = {\text{C}}_{+} \times {\text{m}}_{+}$$

3 $${\text{q}}_{-} = {\text{C}}_{-} \times {\text{m}}_{-}$$

4 $${\text{m}}_{+}/{\text{m}}_{-} = {\text{C}}_{-}/{\text{C}}_{+}$$

where, m + and m- denote the positive (2 mg) and negative (6 mg) electrode masses, and C_+_ and C_−_ denote the positive and negative electrode capacity. The device’s selection of cellulose as the separator and KOH as the electrolyte results in high ionic conductivity, environmental sustainability, and compatibility with both metal sulfide and carbon-based electrode materials, all of which contribute to its high performance and long-term stability. The specified capacities of the rGO material (120 F g^−1^ at 2 A g^−1^) was determined. The mass ratio among the positive and negative electrodes was found to be 2.16 for the best electrochemical performance of ASC. The following formulas were used to calculate the energy and power densities^40^.

5 $${\text{E}} = {\text{CV}}^{2}/7.2.$$

6 $${\text{P}} = {\text{E}}\times 3600/t$$

where, Δt refers the discharging process (s), ΔV represents the potential range (V), the density of energy (E), the density of power (P), and C is the specific capacitance (F g^−1^).

## Result and discussion

**Fig. 1:**
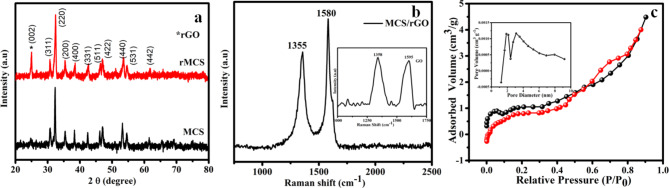
MCS and rMCS (**a**) XRD patterns (**b**) Raman spectrum (insect) GO (**c**) N_2_ adsorption and desorption curve and (insect) pore size distribution profile of sample.

Figure 1a reveals the XRD pattern of MCS & rMCS NPs. The XRD patterns explain diffraction peaks at 2θ values of 30.72°, 32.21°, 35.43°, 38.38°, 42.40°, 46.16°, 47.05°, 53.22°, 54.41° and 61.23° can be indexed to (311), (220), (200), (400), (331), (511), (422), (440), (531) and (442) planes of cubic Co_3_S_4_ (JCPDS card no:73-1703)^35^. Mn is substituted at the Co site of Co_3_S_4_ in MnCoS. Therefore, adding the Mn element only modifies the crystal parameter of MnCoS rather than changing the crystal structure. The obtained peaks show a small shift to the lower 2θ angle, making them more akin to the same system. This could be because Mn^2+^ (0.81 Å) ions are marginally bigger than Co^3+^ (0.68 Å) ions; as a result, the 2θ value is shifted to a lower angle and the *d* spacing is increased^41,42^. The peak at about 25° belongs to the (002) plane of rGO^32^. From XRD analysis, higher crystalline often correlates with better electrochemical performance due to the enhanced structural stability and efficient ion transport pathways. Also, phase purity of certain crystallographic planes might influence the ion diffusion pathways that enhance the overall performance of the electrode materials. Figure 1b reveals the Raman spectrum for the rMCS sample displays two characteristic peaks commonly observed in carbon materials including the D and G mode at 1355 cm^−1^ and 1600 cm^−1^, respectively. The peak at 1355 cm^−1^ signifies the symmetry and formation of carbon crystals, ascribed to surface defects, simultaneous resonant method close to the K point of the Brillouin Zone (BZ) border, and *sp3*-carbon-atom motions within the disarrangement carbon graphitic structure (A_1g_ symmetry at graphene layer edges). On the other hand, the first-order E_2g_ phonon scattered of the *sp*^2^ carbon–carbon union is correlated with the G peak at 1586 cm^−1^. The estimated ID/IG concentration fraction for the rGO/MCS composite stands at 0.85, indicative of the level of organization or randomness within the carbon constitutes. This suggests that sulfurization helps to lower the oxygen type of GO, but high sulfur concentrations can also result in more defect^3,35^. Figure 1b (Insect) shows the Raman spectra for GO, the peaks appear at 1358 cm^−1^ and 1595 cm^−1^ corresponds to D and G band^43^. Using the N_2_ adsorption-desorption isothermal technique; we investigated the SSA and pore size distribution of the arranged rMCS materials are shown in Fig. 1c. A hysteresis loop of adsorption-desorption isotherm profiles at relative pressure (P/P_0_) ranges from 0.5 to 0.9, suggesting a typical isotherm of IV-type and H3-type, indicating the mesoporous structure of the materials. The inset of Fig. 1c depicts the total pore volume and pore size distributions of the sample from BJH process. The prepared sample has a specific surface area (SSA), total pore volume and pore diameters are 32.6 m^2^g^−1^, 0.064 cm^3^g^−1^ and 3.1 nm, respectively. The mesoporous structure and higher surface area significantly improve electrochemical performance by increasing the available active sites for reactions and enhancing ion and electrolyte access. The mesopores facilitate faster ion diffusion; while a larger surface area promotes better charge storage, leading to improved specific capacitance and cycling stability for the MCS and rCMS electrodes^44^. It was practical that the large SSA can offer more surface reaction locate for the electrochemical reaction, thereby greatly improving the electrochemical performance.

**Fig. 2:**
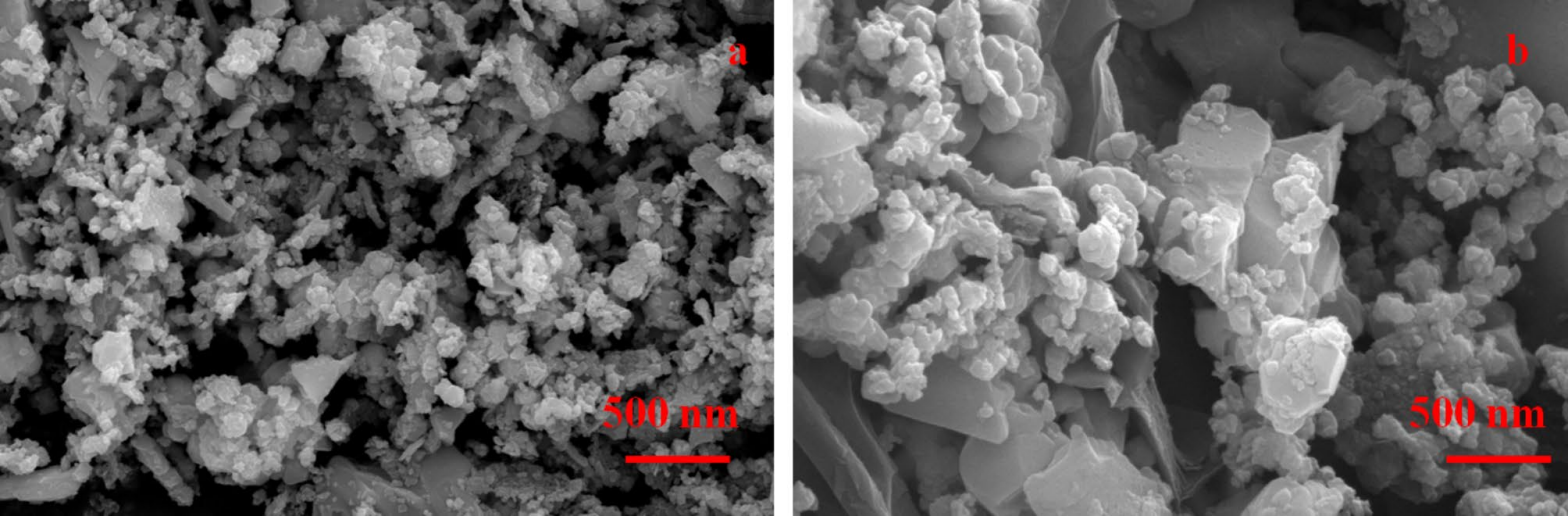
FESEM picture of (**a**) MCS and (**b**) rMCS.

**Fig. 3:**
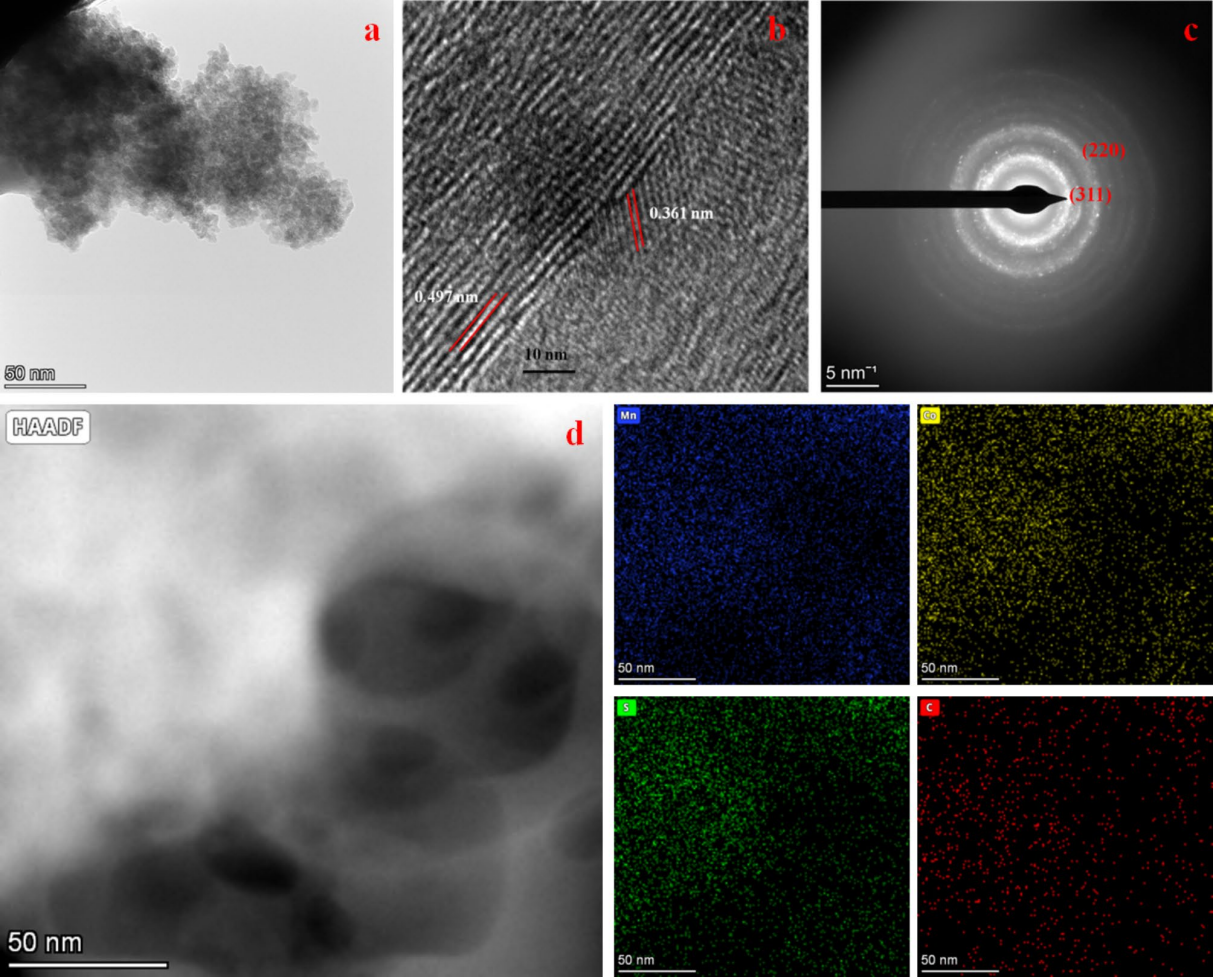
(**a**) TEM image, (**b**) HRTEM, (**c**) SAED pattern, (**d**) STEM and corresponding elemental mapping of rMCS NPs.

Figure 2a,b reveals the FESEM images of MCS and rMCS. The sample shows the nanoparticles have a dimension of around 75 nm (Fig. 2a). Nanoparticles decorated on sheets are seen in Fig. 2b. Due to their large surface area, which offers ions many electroactive reaction sites during an electrochemical reaction, NPs are well known for improving the electrochemical properties of SCs. The nanoparticles typically enhance electrochemical performance by providing a higher surface area-to-volume ratio that improves MCS and rMCS electrodes exposure for electrochemical reactions. Their small size also shortens ion diffusion paths, facilitating faster charge/discharge rates and enhances the electrochemical performance of the electrodes. Figure 3a exhibits the TEM image of the prepared rGO-MCS nanoparticles is much denser with better distribution. Uniformly distributed and well-defined nanoparticles often lead to better performance by enhancing ion diffusion and electron transport. Additionally, smaller particles with controlled morphology can provide more active sites for electrochemical reactions, improving overall specific capacitance and charge/discharge rates. HR-TEM image confirms the lattice rings are about 0.497 nm and 0.361 nm, conspicuously that corresponds to (220) and (002) crystal plane of MCS intimately full-grown on rGO (Fig. 3b). Selected area of electron diffraction (SAED) pattern Fig. 3c imitates the polycrystalline scenery of MCS with bright fringes matching to (311) and (220) planes that matched with XRD results. Figure 3d illustrates the high angle annular dark field scanning transmission electron microscopy (HAADF-STEM) figures of rMCS, proving the homogenous division of Mn, Co, S and C elements.

**Fig. 4:**
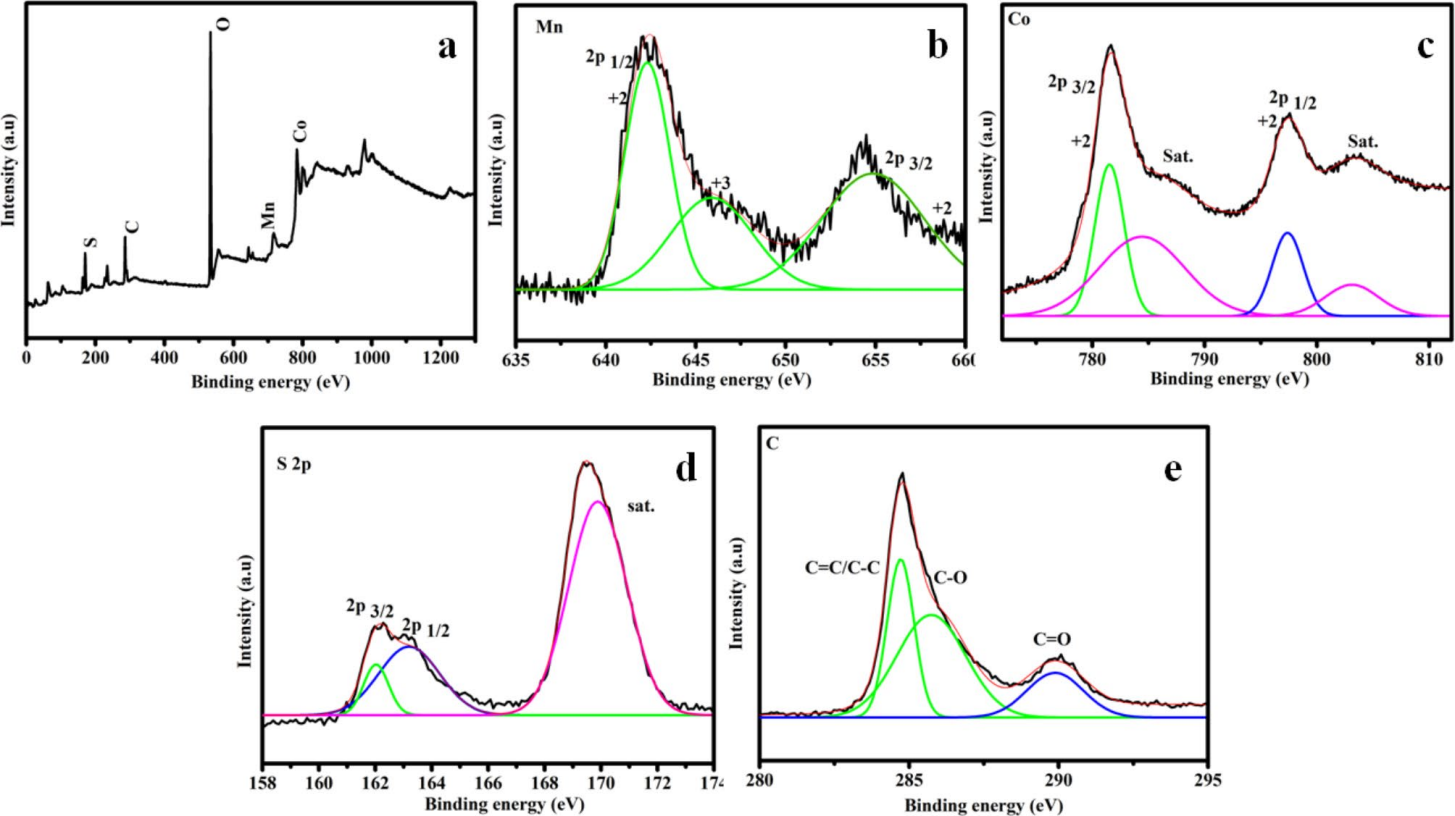
XPS spectra and high resolution spectra of rMCS (**a**) full survey, (**b**) Mn *2p*, (**c**) Co *2p*, (**d**) S *2p* and (**e**) C *1s*.

Figure 4a exhibits the XPS survey of rMCS validating the occurrence of Mn, Co, S and C elements respectively. Figure 4b presents Mn *2p* band, two peaks appear at 642.3 eV and 654.5 eV are allocated to Mn^2+^ and the peak at 646.3 eV are indexed to Mn^3+^.^35^ Co *2p* can be fitted with peaks of 781.5 eV and 797.3 eV are attributed to Co^2+^. Further two peaks at 787.1 eV and 804.1 eV are correspond to the satellite peak (Fig. 4c)^34^. Figure 4d explains the S *2p* band, and the two peaks are to be found at 161.9 eV and 163. 1 eV are assigned to metal sulfur bonding to (S^2−^) ions^45^. The major peak in the C *1s* band at 284.2 eV indicates that rGO is the cause of the existence of C elements that are allotted to C=C/C–C. Additionally, the peaks at 286.18, and 288.18 eV match to C–O, and C=O are listed in Fig. 4e^3,34^. Observing the surface chemistry and the electronic structure of MCS and rCMS electrodes help to predict how well the electrodes can facilitate the charge transfer and maintain stability during cycling. For instance, favorable oxidation states identified in XPS can lead to enhanced conductivity, and cycling stability that directly improving the electrochemical efficiency of the electrodes.

**Fig. 5:**
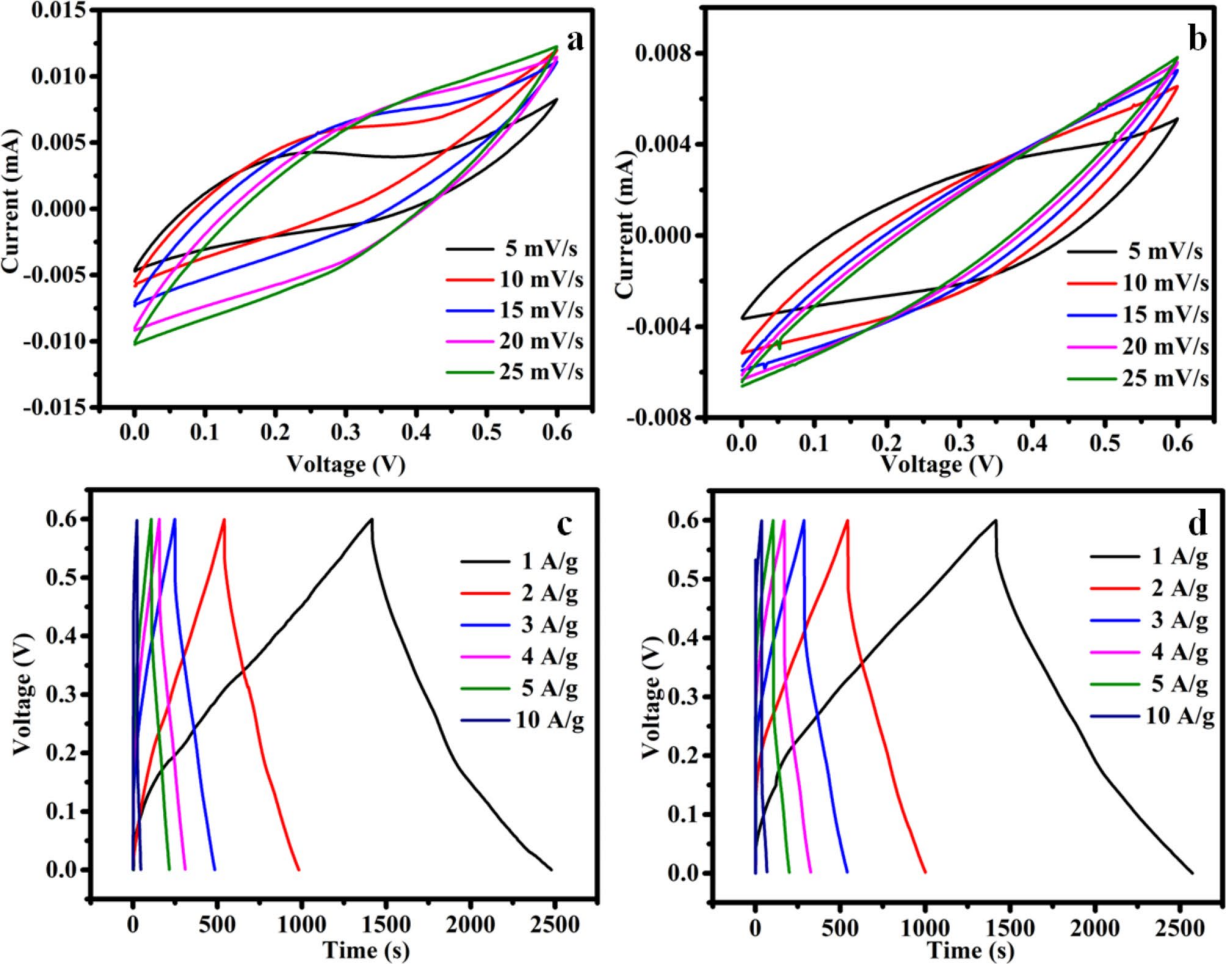
CV and GCD profile for (**a**) MCS at different scan speed, (**b**) rMCS at various scan rate, (**c**) MCS at dissimilar current density and (**d**) rMCS at dissimilar density of current.

The CV curves for MCS and rMCS electrodes at different scan from 5 to 25 mV/s at the voltage range of 0–0.6 V are showed in Fig. 5a,b that express the faradic storage mechanism representing the occurrence of redox reaction during the electrochemical route. As the scan rate an increase, the area under the CV loop expands while the shape of CV profile remains unchanged, suggesting a highly reversible Faradic reaction. The possible redox reaction behavior among the ions of Mn^2+^/Mn^3+^ and Co^2+^/Co^3+^ can be explained as follows^34^.

7 $${\text{CoS}} + {\text{O}}{{\text{H}}^ - } \leftrightarrow {\text{CoSOH}} + {{\text{e}}^ - }$$

8 $${\text{CoSOH }} + {\text{ O}}{{\text{H}}^ - } \leftrightarrow {\text{CoS}} + {{\text{H}}_{\text{2}}}{\text{O}} + {{\text{e}}^ - }$$

9 $${\text{MnS }} + {\text{ O}}{{\text{H}}^ - } \leftrightarrow {\text{MnSOH }} + {\text{ }}{{\text{e}}^ - }$$

The charge storage mechanism in MCS largely involves faradaic redox reactions. Transition metal sulfides, such as MCS, are known for their high electrochemical activity due to the multiple oxidation states of manganese (Mn) and cobalt (Co). These redox reactions occur during the charge/discharge process, with Mn and Co undergoing the electron transfer. This faradaic behavior typically leads to higher specific capacitance compared to purely electrostatic (EDLC) materials like carbon-based electrodes. Inconsistencies in observing faradic processes in MCS and rCMS electrodes may stem from a combination of material and system factors such as poor conductivity, insufficient surface area, or synthesis-related issues. When MCS is combined with reduced graphene oxide (rGO), the charge storage mechanism is enhanced. While MCS maintains its faradaic redox activity, the rGO contributes to the electric double-layer capacitance (EDLC). rGO offers high surface area, excellent electrical conductivity, and abundant defects or functional groups that can store charge via EDLC mechanisms. This hybrid combination results in synergistic effects, improving both charge storage efficiency and electrical conductivity^46^. Figure 5c,d present the GCD curve for MCS and rMCS electrodes under the various density of current varying as of 1 to 10 A g^−1^. GCD profiles validate the presence of distinct pleatues, affirming the occurrence of a Faradic reaction throughout the charging and discharging process. The GCD measurement is reliable with the CV measurement. In comparison to the rMCS and MCS electrode, it is evident from the result that the rMCS electrode essential the highest discharge period. This suggests that rMCS had the highest specific capacity. The ion diffusion in pure MCS materials is somewhat limited by the inherent crystalline and structure of the metal sulfides. MCS typically has a relatively high charge storage capacity, but its structure may present bottlenecks for electrolyte ion diffusion, which could slow down the electrochemical response, especially during high-rate charge/discharge cycles^47^. The inclusion of rGO into MCS significantly enhances ion diffusion. GO has an interconnected porous network with high specific surface area, and when reduced to rGO, it also has improved conductivity. Since the linked structure of rGO allows electrolyte ions to diffuse through the material more efficiently, its presence in the MCS improves ion transport channels. Additionally, it expands the surface area by offering additional EDLC and redox reaction active sites, which expedites the transfer of charges. Because rGO offers a highly conductive matrix that facilitates fast electron mobility across the material during redox reactions, its insertion also lowers charge transfer resistance. rGO helps disperse the MCS particles more evenly, preventing agglomeration and exposing more active sites. Additionally, the flexible and robust structure of rGO prevents mechanical degradation of the electrode during charge/discharge cycles, improving cycling stability and capacitance retention^48,49^.

**Fig. 6:**
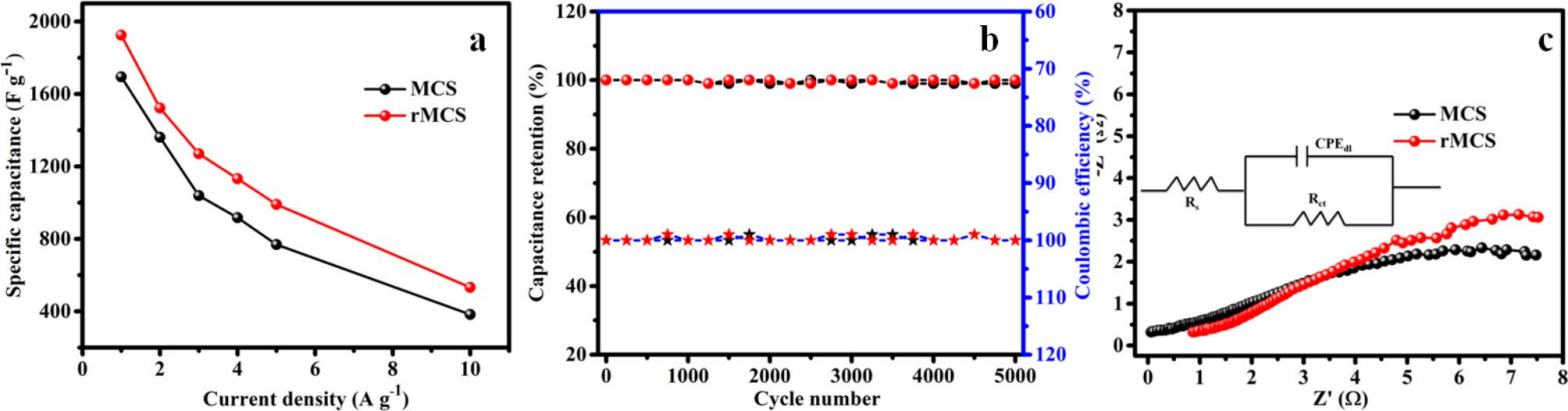
MCS and rMCS of (**a**) specific capacitance at different current densities, (**b**) capacitance retention and coulombic efficiency as a function of cycle number and (**c**) Nyquist plot.

From the GCD profile, the rate performance as a role of various current densities for the prepared electrode is depicted in Fig. 6a. The evaluated high rate performance values for the MCS electrodes is 1695, 1362, 1038.3, 916.6, 768.3 and 383.3 F g^−1^ for different density of current 1, 2, 3, 4, 5 and 10 A g^−1^, correspondingly. The evaluated rate performance for the rMCS electrodes is 1925, 1523.3, 1270, 1133.3, 991.6 and 533.3 F g^−1^ for different densities of current 1, 2, 3, 4, 5 and 10 A g^−1^, correspondingly while the density of current increasing the specific capacitance values decreased. The amalgamation of MCS’s faradaic redox activity and rGO’s electric double-layer capacitance yields rMCS’s higher electrochemical performance over MCS. Higher specific capacitance and quicker charge/discharge rates are the results of this hybrid structure’s improved conductivity, surface area, and ion diffusion. rGO plays a critical role in enhancing the conductivity of composite electrodes due to its inherent high electrical conductivity. The reduction process in graphene oxide removes oxygen-containing groups and restores the conjugated network, thereby enhancing electron mobility across the graphene sheets. This leads to an efficient electron transfer pathway, which is crucial in electrochemical devices, as it facilitates rapid charge and discharge cycles. Also, rGO enhances the electrochemical stability of the composite electrode due to its mechanical robustness and chemical resilience. Its 2D structure provides structural support, preventing the degradation or detachment of active materials during electrochemical cycling. This structural stability minimizes the volume changes during charge/discharge processes, leading to longer cycle life and improved capacity retention^50,51^. By allowing the electrolyte ions to gradually disperse into the interior pores of the electrode surface, reduced current density optimizes the active surface areas of the electrode materials. Only at high densities of current can the electrolyte ions make it to the electrode materials’ surfaces. In order to optimise their active surface areas, the electrode materials do not have enough time to intercalate the inner pores of the electrode surfaces. The retention of capacitance and Coulombic efficiency of the prepared electrode are listed in Fig. 6b. The material exhibits 99% and 100% capacitance retention for MCS and rMCS even after 5000 cycles at the density of current 10 A g^−1^. The electrodes consistent 100% Coulombic efficiency demonstrated the charge storage mechanisms strong reversibility. The robust cyclic stability of the MCS and rMCS electrodes could be attributed to the unique structure of MCS and its composite with reduced graphene oxide. The spinel structure of MCS is known for its high crystallinity and structural stability, which may help prevent lattice distortion or collapse during cycling^52^. The existence of strength implies to an extremely reversible exterior redox reaction occurred on the electrode and electrolyte surfaces. As a result, the rMCS electrode responds to this by having a higher electrochemical conductivity than the MCS electrode.

Figure 6c displays the Nyquist plot for MCS and rMCS electrodes with the frequency vary of 0.01 Hz to 100 kHz. A vertical line appears in the low-frequency region and a tiny semicircle appears in the high-frequency region according to the Nyquist plot. The solution resistance is found by tracing the real axis intercept at impedance. The prepared electrode has the solution resistance (R_s_) of 0.5 Ω for MCS and 0.9 Ω for rMCS that signifies the improved conductivity of the prepared rMCS electrode material. The charge transfer resistance (R_ct_) at the electrode/electrolyte boundary is given by the radius of the semicircle part. The 45° slope line, also known as the Warburg line, is seen at the low-frequency part and is connected to the real axis. This is caused by the ion’s resistance to diffusion in the electrolyte. The slope line at 45° indicates that the electrode charge transfer system is controlled by diffusion rather than being pure capacitive^41^.

**Fig. 7:**
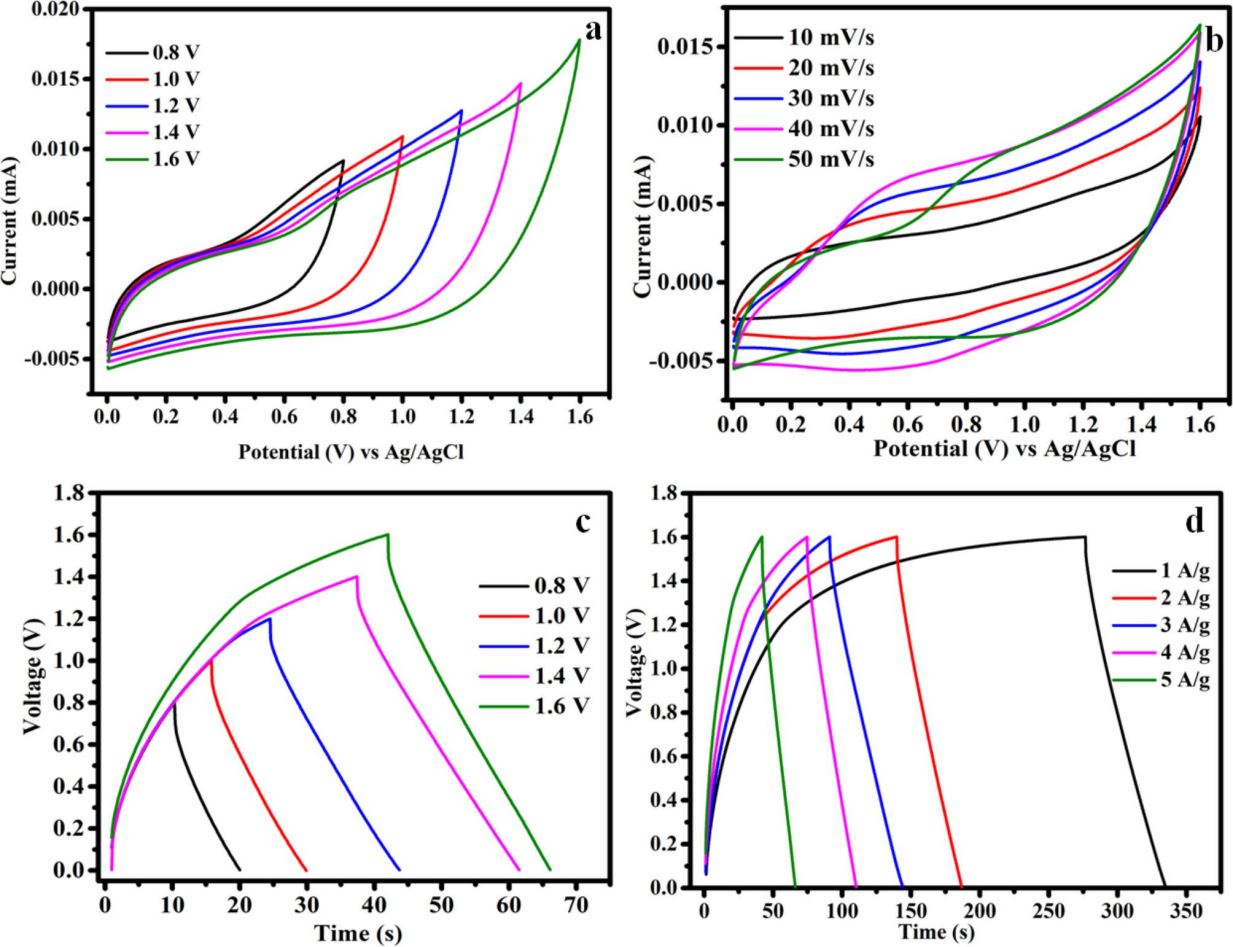
CV and GCD curve for rMCS//rGO the fabricated machine (**a**) at dissimilar potential, (**b**) at various scan rate at cell voltage of 1.6 V, (**c**) at various potential and (**d**) at various densities of current at cell voltage of 1.6 V.

Fabricated device rMCS//rGO CV curves at various cell voltage from 0.8 to 1.6 V at scan speeds of 50 mVs^−1^ is given in Fig. 7a. The machine reveals the peaks performance inside the potential of 0–1.6 V. The CV profile of rMCS//rGO at different scan rate be listed in Fig. 7b. It be supposed to be mentioned that redox peak are also visible in the cells of CV profile. The cells CV curve below rising scan rates exhibits a movement that is comparable to the positive electrode materials. The GCD curve at various cell potential ranging from 0.8 to 1.6 V with the density of current 5 A g^−1^ is shown in Fig. 7c. The GCD curves show almost symmetrical shape indicates the devices superior electrochemical reversibility. Figure 7d shows the GCD curve of the fabricated device at 1.6 V cell potential and different density of current. The discharge duration decreases clearly as the current density is increased. The insufficient redox reaction of the material at high densities of current is the reason for this phenomenon.

**Fig. 8:**
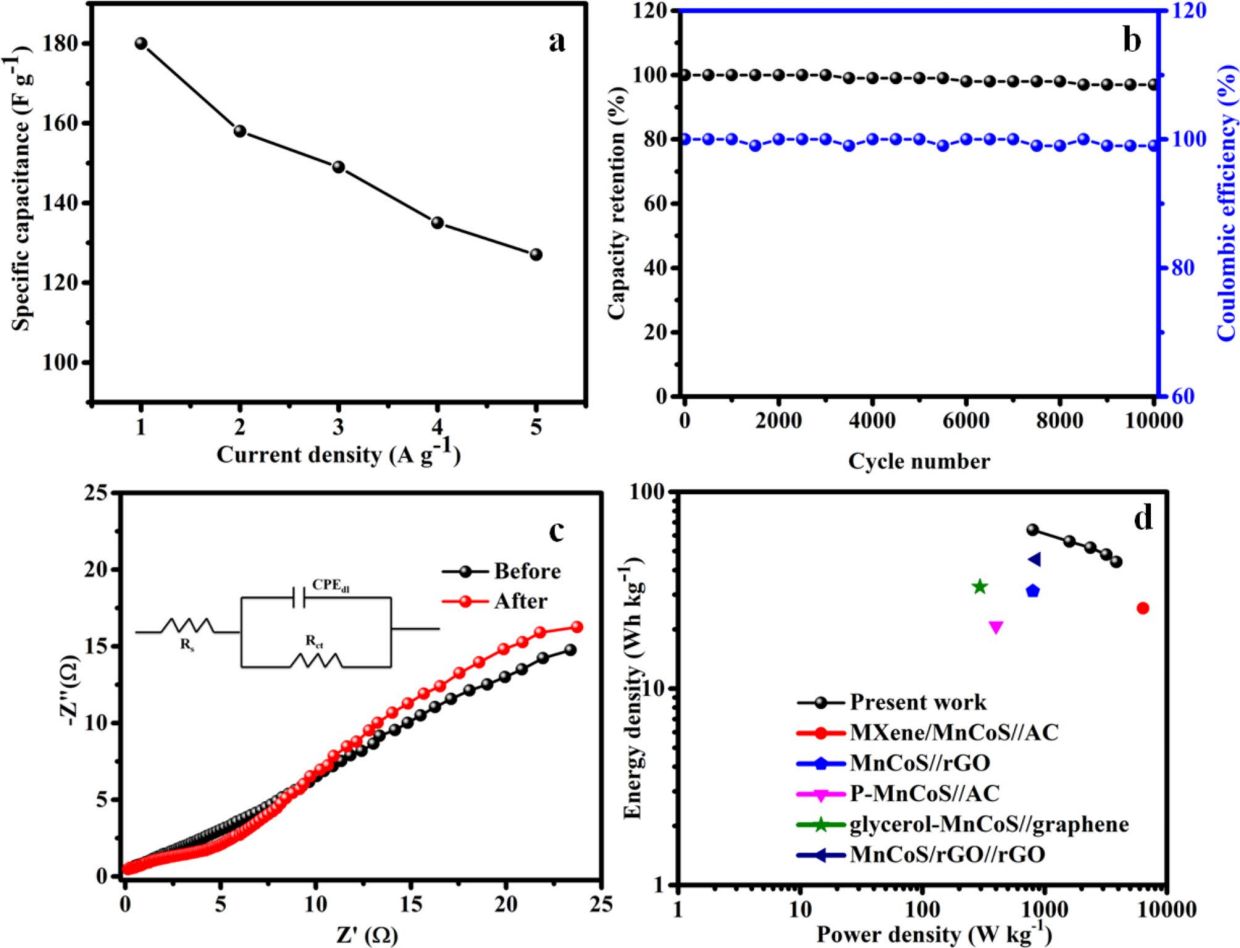
Fabricated rMCS//rGO (**a**) changes in specific capacitance with respect to density of current, (**b**) retention capacitance and Coulombic efficiency vs. cycle number, (**c**) Nyquist plot before and after cycle and (**d**) Ragone plot.

The plot of the rate performance vs. current densities is worn to learn the rate performance of the prepared material is given in Fig. 8a. The specific capacitance values evaluated from GCD profile are 180, 157.5, 148.1, 135 and 128.1 Fg^−1^ at the densities of current 1, 2, 3, 4 and 5 A g^−1^, correspondingly. Figure 8b reveals the 97.4% of cyclic stability up to 10,000 cycles at the densities of current 10 A g^−1^. In adding, the fabricated device displays an outstanding Coulombic efficiency of 99% upto 10,000 cycles. By providing more conductive routes and a more porous structure, the rGO sheets in rMCS probably speed up ion diffusion and lessen the possibility of ion entrapment or depletion zones inside the electrode material. This guarantees that during the cycling process, the electrolyte may interact with the active sites in an efficient manner. The graphene matrix can prevent the agglomeration of MCS particles and maintain structural integrity during volume changes caused by ion intercalation/deintercalation. This mechanical robustness provided by rGO helps to retain the active material’s structural and electrochemical performance, leading to better cyclic stability. The previous reported electrochemical behaviors of the asymmetric supercapacitor are tabulated in Table 1. The fabricated rMCS//rGO device shows the Nyquist plot with the frequency vary of 0.01 Hz to 100 kHz (Fig. 8c). The R_s_ and R_ct_ value of the fabricated rMCS//rGO device are obtained as 0.4 Ω and 3.8 Ω, respectively which indicates that it has a high electrical conductivity. Figure 8d shows the Ragone plot for the made-up device gives a density of energy and power of 64 W h kg^−1^ and 799 W kg^−1^. Still at density of power increases to 3862 W kg^−1^ the cell is capable to attain the density of energy 44 Wh kg^−1^, demonstrating a strong rate of performance. The rMCS//rGO devices has an energy and power density ideals higher than those of earlier studies using the same kind of electrode materials. The density of energy and power are compared with already reported MnCoS based asymmetric supercapacitor materials of MnCoS/rGO//rGO (45.4 Wh kg^−1^ at 850 W kg^−1^)^31^, MnCoS/MXene//AC (25.6 Wh kg^−1^ at 6400 W kg^−1^)^42^, MnCoS//rGO (31.3 Wh kg^−1^ at 800 W kg^−1^)^53^, P-MnCoS//AC (20.8 Wh kg^−1^ at 400 W kg^−1^)^54^, glycerol-MnCoS//graphene (32.9 Wh kg^−1^ at 295.2 W kg^−1^).^55^

**Table 1:** Comparison study of rMCS//rGO based asymmetric supercapacitor device with previous report.

Electrode	Potential window	Specific capacitance	Cyclic stability	References
MnCoS@NF//rGO@NF	0–1.5 V	1952 F g^−1^ @ 2 Ag^−1^	93% (3000 cycles)	^22^
MnCo_2_S_4_//rGO/Ni	0–1.6 V	88 F g^−1^ @ 1 Ag^−1^	89% (5000 cycles)	^53^
MnCo_2_S_4_//AC	0–1.6 V	52.3 Fg^−1^ @ 1 Ag^−1^	91.1% (5000 cycles)	^54^
MnCo_2_S_4_//graphene	0–1.5 V	132 F g^−1^ @ 2 A g^−1^	87.7% (5000 cycles)	^55^
ZnCoS//AC	0–1.4 V	79.4 Fg^−1^ @ 1 Ag^−1^	97% (1000 cycles)	^56^
CoMoS_4_//AC	0–1.0 V	68 Fg^−1^ @ 1 Ag^−1^	86% (10,000 cycles)	^57^
PPy/FeCoS-rGO//rGO	0–1.4 V	94 Fg^−1^ @ 1 Ag^−1^	97.5% (2500 cycles)	^58^
MnCo_2_S_4_//rGO	0–1.8 V	162 Fg^−1^ @ 1 Ag^−1^	99% (2500 cycles)	^41^
rMCS//rGO	0–1.6 V	180 Fg^−1^ @ 1 Ag^−1^	97% (2500 cycles)	Present work

Below is a summary of the diverse factors contributing to the outstanding electrochemical characteristics of rMCS electrodes: (i) the capability of rMCS nanoparticles to facilitate ion transfer pathway during electrochemical reactions (ii) A large specific surface area of the nanoparticles, offering ample surface reaction sites. (iii) Enhancement of electrical conductivity by Mn and Co ions within the electrodes. (iv) A pronounced interdependent effect of Mn and Co ions leading to significant enhancements in electrochemical performance.

## Conclusion

In summary, we successfully prepared MCS and rMCS NPs via hydrothermal method for high performance supercapacitor applications. From the morphological studies we confirm the nanoparticles with a size of 75 nm. BET results exhibit high surface area of the arranged materials. The composite material displays a high specified capacity and cyclic strength in the 3-electrode configurations. The fabricated asymmetric supercapacitor device rMCS//rGO shows the specified capacitance of 180 F g^−1^ at 1 A g^−1^ with an initial capacitance of 97.4% up to 10,000 cycles. The assembled device delivers a density of energy 64 Wh kg^−1^ at the density of power 799 W kg^−1^. Even as a density of power increases by 3862 W kg^−1^ the cell was capable to reach a density of energy 44 Wh kg^−1^. The MnCoS is less extensive because of the rGO, which also offers a quick electron transport channel. As a result, the material’s cyclic stability during the charge and discharge process was greatly enhanced. The prepared materials validate the promising potential for next-generation high-performance supercapacitors.

## Data Availability

All the data are included in the manuscript.
